# Supplementary material for: Effects of β-Glucan Supplementation on LPS-Induced Endotoxemia in Horses
Source: Animals (Basel). 2024 Jan 31;14(3):474. doi: 10.3390/ani14030474 (PMC10854761; doi:10.3390/ani14030474)
Supplement: Supplementary file 1 [file animals-14-00474-s001.zip › ELISA assays graphics- Supplement.pdf]

Four-parameter.log(2.74, 9.03, 0.54, 6.12)

DC=(1.17,45074.55) Chi=8.17%

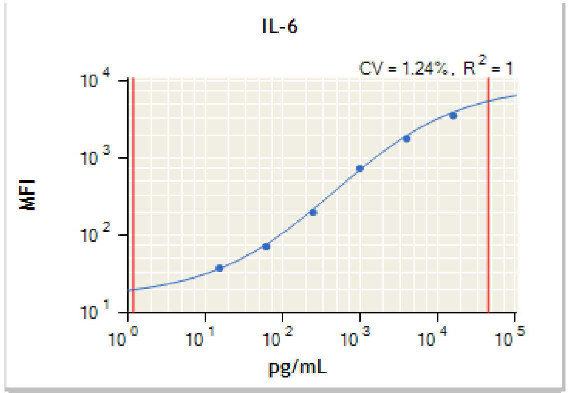

Four-parameter.log(2.62, 8.64, 0.53, 6.69)

DC=(2.10,835390.10) Chi= 2.86%

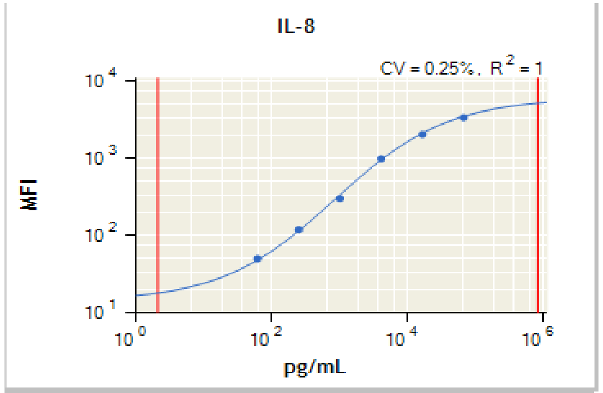

Four-parameter.log(3.57, 8.55, 0.80, 7.16)

DC=(4.98,89107.53) Chi=9.76%

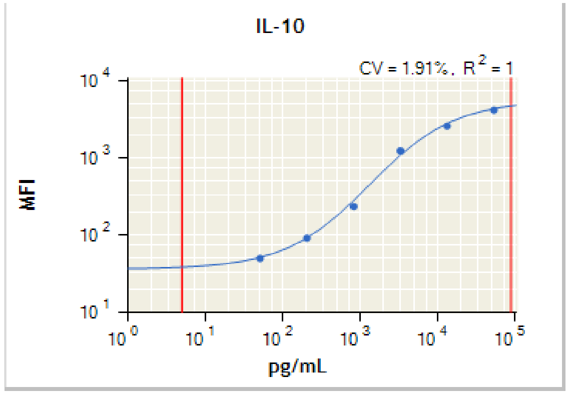

Y = Linear({1.36, 3.5}, {8.29, 8.5})

DC=(,+∞) Chi=

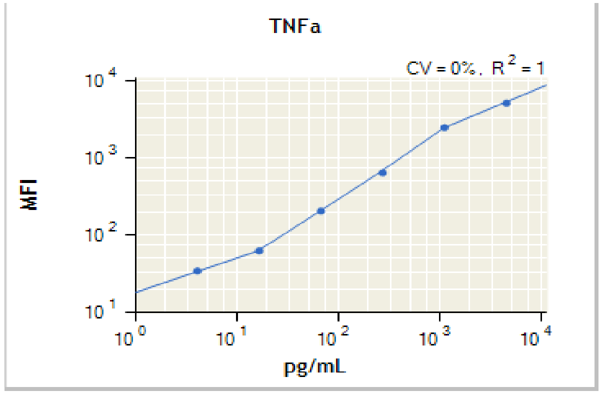

Files Name:

C:\Documents and Settings\Administrator\Desktop\Copy of Brunetto-EQCYTMAG-93K.csv

Chip Model: LINCOplex

Application Version: 3.5.5.0

User Name: Administrator

Signature:

Organization: LEAC-Lab. Especializado em Análieses Científicas
