# Supplementary material for: Effects of β-Glucan Supplementation on LPS-Induced Endotoxemia in Horses
Source: Animals (Basel). 2024 Jan 31;14(3):474. doi: 10.3390/ani14030474 (PMC10854761; doi:10.3390/ani14030474)
Supplement: Supplementary file 1 [file animals-14-00474-s001.zip › ELISA assays IL-10 - Supplement.pdf]

Analyte IL-10

Four-parameter.log(3.57, 8.55, 0.80, 7.16)

Chi=9.76%, CV=1.91%, R2=1.00, DC=(4.98, 89107.53)

Expected pg/mL (i)	MFI(i)	MFI	CV	pg/mL(i)	pg/mL	Recovery
0	16.5	17	4.16%	0	0	
	17.5			0		
48.83	50	50		48,56	48.56	99 %
	50			48,56		
195	91	91		206	206	106 %
	~~97~~			229		
781	235	235		691	691	89 %
	~~231~~			679		
3125	~~1197~~	1152.5		3826	3656	117 %
	1152.5			3656		
12500	2484	2479.5	0.26%	11451	11410	91 %
	2475			11369		
50000	3966.5	4041.75	2.63%	45986	50591	101 %
	4117			55942		

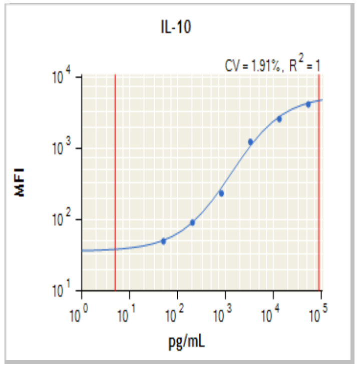

Samples:

Sample		MFI(i)	MFI	CV	pg/mL(i)	pg/mL	
Control1		108.5	109.25	0.97%	271	273	
		110			276		
Control2		773	776.5	0.64%	2338	2350	
		780			2361		
1		64	64		103	103	

3	103	103	251	251
4	133	133	357	357
5	164	164	462	462
6	313.5	313.5	933	933
7	221	221	647	647
8	316	316	940	940
9	428	428	1277	1277
10	333.5	333.5	993	993
13	128.5	128.5	341	341
14	64	64	103	103
16	123	123	322	322
17	161.5	161.5	454	454
18	122.5	122.5	320	320
19	295	295	877	877
20	306	306	910	910
21	316	316	940	940
22	433.5	433.5	1294	1294
23	368	368	1097	1097
26	83.5	83.5	178	178
27	51	51	52,43	52.43
29	48	48	40,9	40.9
30	837	837	2546	2546
31	64	64	103	103
32	100	100	240	240
33	202.5	202.5	588	588
34	168	168	476	476

35	235	235	691	691
36	283	283	840	840
39	94	94	218	218
40	149	149	412	412
42	53.5	53.5	62,13	62.13
43	71	71	130	130
44	121	121	315	315
45	151	151	419	419
46	238	238	701	701
47	345	345	1028	1028
48	423.5	423.5	1264	1264
49	423	423	1262	1262
52	85	85	184	184
53	45.5	45.5	31,47	31.47
55	42	42	18,73	18.73
56	39	39	8,65	8.65
57	45	45	29,61	29.61
58	99	99	236	236
59	184	184	528	528
60	248	248	732	732
61	229	229	672	672
62	175.5	175.5	500	500
65	79	79	161	161
66	47	47	37,1	37.1
68	45	45	29,61	29.61
69	42.5	42.5	20,51	20.51

70	48	48	40,9	40.9
71	119	119	308	308
72	117	117	301	301
73	146.5	146.5	403	403
74	215	215	628	628
75	150	150	415	415
78	123.5	123.5	324	324
79	117	117	301	301
81	121	121	315	315
83	165.5	165.5	467	467
84	87	87	191	191
85	369.5	369.5	1102	1102
86	276	276	818	818
87	243.5	243.5	718	718
88	290	290	861	861
91	121	121	315	315
92	57	57	75,8	75.8
94	178	178	509	509
96	155.5	155.5	434	434
97	288	288	855	855
98	233.5	233.5	686	686
99	308	308	916	916
100	215	215	628	628
101	396	396	1181	1181
104	171.5	171.5	487	487

Notes: Red-Above range, Black-Below range, Blue-In range
