# Supplementary material for: Effects of β-Glucan Supplementation on LPS-Induced Endotoxemia in Horses
Source: Animals (Basel). 2024 Jan 31;14(3):474. doi: 10.3390/ani14030474 (PMC10854761; doi:10.3390/ani14030474)
Supplement: Supplementary file 1 [file animals-14-00474-s001.zip › ELISA assays IL-6 - Supplement.pdf]

Analyte IL-6

Four-parameter.log(2.74, 9.03, 0.54, 6.12)

Chi=8.17%, CV=1.24%, R2=1.00, DC=(1.17, 45074.55)

Expected pg/mL (i)	MFI(i)	MFI	CV	pg/mL(i)	pg/mL	Recovery
0	15.5	*14.75*	7.19%	0	0	
	14			0		
14.65	~~38~~	36		16,7	14.62	100 %
	36			14,62		
58.59	71.5	74.5	5.69%	56,19	59.96	102 %
	77.5			63,76		
234	196	191.75	3.13%	222	216	92 %
	187.5			210		
938	718	735.25	3.32%	1045	1076	115 %
	752.5			1108		
3750	1719	1690	2.43%	3444	3354	89 %
	1661			3265		
15000	3679	3679		15331	15331	102 %
	3679			15331		

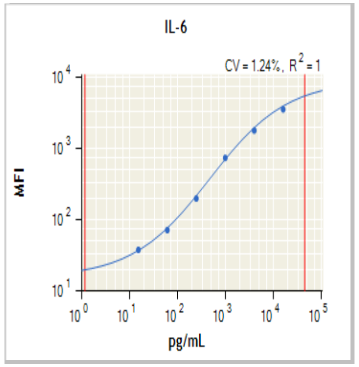

Samples:

Sample	MFI(i)	MFI	CV	pg/mL(i)	pg/mL
Control1	99.5	99.25	0.36%	92,08	91.76
	99			91,43	
Control2	618	611.25	1.56%	870	858
	604.5			847	
1	24	24		3,83	3.83

3	25	25	4,58	4.58
4	34.5	34.5	13,1	13.1
5	38	38	16,7	16.7
6	60	60	41,99	41.99
7	76	76	61,86	61.86
8	159	159	171	171
9	108	108	103	103
10	51.5	51.5	31,83	31.83
13	28	28	7,01	7.01
14	21	21	1,89	1.89
16	26	26	5,35	5.35
17	37	37	15,65	15.65
18	42	42	21,01	21.01
19	142	142	148	148
20	163	163	177	177
21	262	262	315	315
22	205	205	235	235
23	150.5	150.5	160	160
26	33	33	11,62	11.62
27	22	22	2,48	2.48
29	23	23	3,14	3.14
30	758	758	1118	1118
31	28.5	28.5	7,45	7.45
32	69.5	69.5	53,69	53.69
33	154	154	165	165
34	375.5	375.5	481	481

35	421	421	551	551
36	343	343	433	433
39	109	109	105	105
40	102.5	102.5	96	96
42	22	22	2,48	2.48
43	23	23	3,14	3.14
44	32.5	32.5	11,13	11.13
45	73.5	73.5	58,71	58.71
46	128	128	130	130
47	375.5	375.5	481	481
48	326	326	408	408
49	295	295	362	362
52	62	62	44,42	44.42
53	20	20	1,35	1.35
55	23	23	3,14	3.14
56	35	35	13,6	13.6
57	21	21	1,89	1.89
58	29	29	7,89	7.89
59	47	47	26,61	26.61
60	175	175	193	193
61	95	95	86,23	86.23
62	48	48	27,76	27.76
65	25	25	4,58	4.58
66	20	20	1,35	1.35
68	25	25	4,58	4.58
69	23	23	3,14	3.14

70	24	24	3,83	3.83
71	73	73	58,08	58.08
72	33	33	11,62	11.62
73	54	54	34,78	34.78
74	67.5	67.5	51,2	51.2
75	36	36	14,62	14.62
78	51	51	31,24	31.24
79	60	60	41,99	41.99
81	61	61	43,2	43.2
83	117	117	115	115
84	58	58	39,56	39.56
85	157	157	169	169
86	141	141	147	147
87	129	129	131	131
88	81	81	68,21	68.21
91	39.5	39.5	18,29	18.29
92	49	49	28,91	28.91
94	163.5	163.5	178	178
96	119	119	118	118
97	127	127	128	128
98	91	91	81,05	81.05
99	130	130	132	132
100	96.5	96.5	88,18	88.18
101	134	134	138	138
104	151	151	161	161

Notes: Red-Above range, Black-Below range, Blue-In range
