# Supplementary material for: Effects of β-Glucan Supplementation on LPS-Induced Endotoxemia in Horses
Source: Animals (Basel). 2024 Jan 31;14(3):474. doi: 10.3390/ani14030474 (PMC10854761; doi:10.3390/ani14030474)
Supplement: Supplementary file 1 [file animals-14-00474-s001.zip › ELISA assays IL-8- Supplement.pdf]

Analyte IL-8

Four-parameter.log(2.62, 8.64, 0.53, 6.69)

Chi=2.86%, CV=0.25%, R2=1.00, DC=(2.10, 835390.10)

Expected pg/mL (i)	MFI(i)	MFI	CV	pg/mL(i)	pg/mL	Recovery
0	11	*10.5*	6.73%	0	0	
	10			0		
58.59	~~50~~	46		68,2	58.1	99 %
	46			58,1		
234	~~117.5~~	111.5		262	243	104 %
	111.5			243		
938	~~295~~	302		864	889	95 %
	302			889		
3750	951.5	903.5	7.51%	4163	3852	103 %
	855.5			3554		
15000	1949	1955	0.43%	14865	14964	100 %
	1961			15064		
60000	3210	3223.5	0.59%	59005	59928	100 %
	3237			60869		

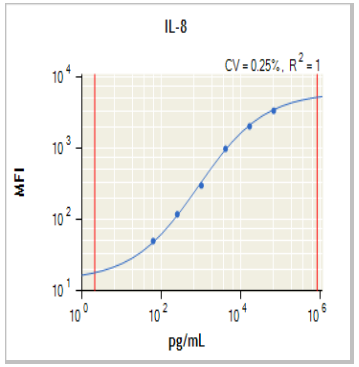

Samples:

Sample		MFI(i)	MFI	CV	pg/mL(i)	pg/mL	
Control1		178	164	12.07%	454	409	
		150			364		
Control2		563.5	563.5		1987	1987	
		563.5			1987		
1		151	151		367	367	

3	157.5	157.5	388	388
4	176.5	176.5	449	449
5	147	147	354	354
6	125.5	125.5	286	286
7	149	149	360	360
8	164	164	409	409
9	152	152	370	370
10	141.5	141.5	336	336
13	149	149	360	360
14	73	73	130	130
16	79.5	79.5	149	149
17	75.5	75.5	137	137
18	58	58	89,14	89.14
19	80.5	80.5	152	152
20	70	70	122	122
21	68.5	68.5	118	118
22	71	71	125	125
23	72.5	72.5	129	129
26	74	74	133	133
27	133	133	310	310
29	120	120	269	269
30	709	709	2717	2717
31	120	120	269	269
32	124	124	282	282
33	128	128	294	294
34	122	122	276	276

35	118	118	263	263
36	106	106	227	227
39	137	137	322	322
40	206.5	206.5	550	550
42	87.5	87.5	172	172
43	99.5	99.5	207	207
44	92	92	185	185
45	84.5	84.5	163	163
46	105	105	224	224
47	172.5	172.5	436	436
48	101	101	212	212
49	189.5	189.5	493	493
52	76	76	139	139
53	60.5	60.5	95,85	95.85
55	65	65	108	108
56	139	139	329	329
57	34	34	29,89	29.89
58	54	54	78,56	78.56
59	61	61	97,2	97.2
60	118	118	263	263
61	53	53	75,95	75.95
62	53	53	75,95	75.95
65	57	57	86,47	86.47
66	46	46	58,1	58.1
68	76	76	139	139
69	80	80	150	150

70	67	67	114	114
71	126	126	288	288
72	53.5	53.5	77,25	77.25
73	61	61	97,2	97.2
74	57	57	86,47	86.47
75	53	53	75,95	75.95
78	51	51	70,77	70.77
79	56	56	83,82	83.82
81	53	53	75,95	75.95
83	116	116	257	257
84	58	58	89,14	89.14
85	54	54	78,56	78.56
86	58.5	58.5	90,47	90.47
87	53	53	75,95	75.95
88	56	56	83,82	83.82
91	47	47	60,6	60.6
92	63.5	63.5	104	104
94	94.5	94.5	192	192
96	60.5	60.5	95,85	95.85
97	53.5	53.5	77,25	77.25
98	47	47	60,6	60.6
99	52.5	52.5	74,65	74.65
100	59.5	59.5	93,15	93.15
101	62	62	99,9	99.9
104	49	49	65,65	65.65

Notes: Red-Above range, Black-Below range, Blue-In range
