# Supplementary material for: Effects of β-Glucan Supplementation on LPS-Induced Endotoxemia in Horses
Source: Animals (Basel). 2024 Jan 31;14(3):474. doi: 10.3390/ani14030474 (PMC10854761; doi:10.3390/ani14030474)
Supplement: Supplementary file 1 [file animals-14-00474-s001.zip › ELISA assays TNF a- Supplement.pdf]

## Analyte TNFa

Y = Linear( {1.36, 3.5}, {8.29, 8.5})

Chi=, CV=, R2=1.00, DC=(, +∞)

| Expected pg/mL (i) | MFI(i) | MFI | CV | pg/mL(i) | pg/mL | Recovery |
|---|---|---|---|---|---|---|
| 0 | 12 | 12 | | 0,41 | 0.41 | |
| | ~~13~~ | | | 0,49 | | |
| 3.91 | ~~34.5~~ | 33 | | 4,32 | 3.91 | 100 % |
| | 33 | | | 3,91 | | |
| 15.62 | 62.5 | 61.25 | 2.89% | 16 | 15.62 | 100 % |
| | 60 | | | 14,91 | | |
| 62.5 | 202 | 197.75 | 3.04% | 64,08 | 62.5 | 100 % |
| | 193.5 | | | 60,91 | | |
| 250 | 625 | 646 | 4.6% | 241 | 250 | 100 % |
| | 667 | | | 259 | | |
| 1000 | 2375 | 2295 | 4.93% | 1065 | 1000 | 100 % |
| | 2215 | | | 962 | | |
| 4000 | 4862 | 4898.5 | 1.05% | 3946 | 4000 | 100 % |
| | 4935 | | | 4055 | | |

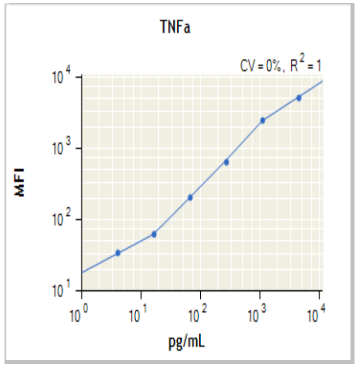


Samples:

| Sample | | MFI(i) | MFI | CV | pg/mL(i) | pg/mL | |
|---|---|---|---|---|---|---|---|
| Control1 | | 51.5 | 54.75 | 8.39% | 10,59 | 12.15 | |
| | | 58 | | | 13,82 | | |
| Control2 | | 328 | 322.5 | 2.41% | 113 | 111 | |
| | | 317 | | | 109 | | |
| 1 | | 22.5 | 22.5 | | 1,66 | 1.66 | |

| | | | | |
|---|---|---|---|---|
| 3 | 2115 | 2115 | 915 | 915 |
| 4 | 3288 | 3288 | 1930 | 1930 |
| 5 | 4185 | 4185 | 3000 | 3000 |
| 6 | 3624 | 3624 | 2305 | 2305 |
| 7 | 1704 | 1704 | 722 | 722 |
| 8 | 572 | 572 | 217 | 217 |
| 9 | 174 | 174 | 53,72 | 53.72 |
| 10 | 59 | 59 | 14,36 | 14.36 |
| 13 | 36 | 36 | 4,75 | 4.75 |
| 14 | 25.5 | 25.5 | 2,19 | 2.19 |
| 16 | 1229.5 | 1229.5 | 505 | 505 |
| 17 | 4238.5 | 4238.5 | 3070 | 3070 |
| 18 | 4549 | 4549 | 3494 | 3494 |
| 19 | 5003.5 | 5003.5 | 4158 | 4158 |
| 20 | 3316.5 | 3316.5 | 1960 | 1960 |
| 21 | 1279.5 | 1279.5 | 528 | 528 |
| 22 | 223 | 223 | 71,94 | 71.94 |
| 23 | 75 | 75 | 19,85 | 19.85 |
| 26 | 33 | 33 | 3,91 | 3.91 |
| 27 | 25 | 25 | 2,1 | 2.1 |
| 29 | 26 | 26 | 2,29 | 2.29 |
| 30 | 994 | 994 | 401 | 401 |
| 31 | 2295 | 2295 | 1000 | 1000 |
| 32 | 5410.5 | 5410.5 | 4797 | 4797 |
| 33 | 4818 | 4818 | 3881 | 3881 |
| 34 | 3836 | 3836 | 2558 | 2558 |

| | | | | |
|---|---|---|---|---|
| 35 | 1942 | 1942 | 833 | 833 |
| 36 | 238 | 238 | 77,64 | 77.64 |
| 39 | 30.5 | 30.5 | 3,28 | 3.28 |
| 40 | 100 | 100 | 27,9 | 27.9 |
| 42 | 136.5 | 136.5 | 40,31 | 40.31 |
| 43 | 514 | 514 | 191 | 191 |
| 44 | 4592 | 4592 | 3554 | 3554 |
| 45 | 4941.5 | 4941.5 | 4064 | 4064 |
| 46 | 4171 | 4171 | 2981 | 2981 |
| 47 | 2334 | 2334 | 1031 | 1031 |
| 48 | 656 | 656 | 254 | 254 |
| 49 | 171 | 171 | 52,63 | 52.63 |
| 52 | 26 | 26 | 2,29 | 2.29 |
| 53 | 22 | 22 | 1,58 | 1.58 |
| 55 | 41 | 41 | 6,36 | 6.36 |
| 56 | 27 | 27 | 2,49 | 2.49 |
| 57 | 111 | 111 | 31,56 | 31.56 |
| 58 | 4183.5 | 4183.5 | 2998 | 2998 |
| 59 | 2294 | 2294 | 1000 | 1000 |
| 60 | 848.5 | 848.5 | 337 | 337 |
| 61 | 179 | 179 | 55,55 | 55.55 |
| 62 | 37 | 37 | 5,05 | 5.05 |
| 65 | 24 | 24 | 1,92 | 1.92 |
| 66 | 20.5 | 20.5 | 1,35 | 1.35 |
| 68 | 22 | 22 | 1,58 | 1.58 |
| 69 | 26 | 26 | 2,29 | 2.29 |

| | | | | |
|---|---|---|---|---|
| 70 | 1188 | 1188 | 487 | 487 |
| 71 | 3577.5 | 3577.5 | 2252 | 2252 |
| 72 | 2349.5 | 2349.5 | 1044 | 1044 |
| 73 | 528.5 | 528.5 | 198 | 198 |
| 74 | 208 | 208 | 66,31 | 66.31 |
| 75 | 151 | 151 | 45,43 | 45.43 |
| 78 | 222 | 222 | 71,57 | 71.57 |
| 79 | 52.5 | 52.5 | 11,06 | 11.06 |
| 81 | 53 | 53 | 11,3 | 11.3 |
| 83 | 1885 | 1885 | 806 | 806 |
| 84 | 2342 | 2342 | 1038 | 1038 |
| 85 | 2433.5 | 2433.5 | 1113 | 1113 |
| 86 | 431 | 431 | 156 | 156 |
| 87 | 132.5 | 132.5 | 38,92 | 38.92 |
| 88 | 74 | 74 | 19,54 | 19.54 |
| 91 | 39 | 39 | 5,68 | 5.68 |
| 92 | 35 | 35 | 4,46 | 4.46 |
| 94 | 131 | 131 | 38,4 | 38.4 |
| 96 | 1816 | 1816 | 774 | 774 |
| 97 | 1588 | 1588 | 669 | 669 |
| 98 | 1418 | 1418 | 591 | 591 |
| 99 | 452 | 452 | 165 | 165 |
| 100 | 95 | 95 | 26,25 | 26.25 |
| 101 | 91 | 91 | 24,95 | 24.95 |
| 104 | 65 | 65 | 16,76 | 16.76 |

Notes: Red-Above range, Black-Below range, Blue-In range
